# Unique yellow shifts for small and brief stimuli in the central retina

**DOI:** 10.1167/jov.24.6.2

**Published:** 2024-06-04

**Authors:** Maxwell J. Greene, Alexandra E. Boehm, John E. Vanston, Vimal P. Pandiyan, Ramkumar Sabesan, William S. Tuten

**Affiliations:** 1 Herbert Wertheim School of Optometry and Vision Science, University of California, Berkeley, Berkeley, CA, USA; 2 Department of Ophthalmology, University of Washington, Seattle, WA, USA

**Keywords:** color vision, unique hues, adaptive optics, cones

## Abstract

The spectral locus of unique yellow was determined for flashes of different sizes (<11 arcmin) and durations (<500 ms) presented in and near the fovea. An adaptive optics scanning laser ophthalmoscope was used to minimize the effects of higher-order aberrations during simultaneous stimulus delivery and retinal imaging. In certain subjects, parafoveal cones were classified as L, M, or S, which permitted the comparison of unique yellow measurements with variations in local L/M ratios within and between observers. Unique yellow shifted to longer wavelengths as stimulus size or duration was reduced. This effect is most pronounced for changes in size and more apparent in the fovea than in the parafovea. The observed variations in unique yellow are not entirely predicted from variations in L/M ratio and therefore implicate neural processes beyond photoreception.

## Introduction

Color science is fundamentally concerned with how spectral information from objects in the environment is encoded by the three types of cone photoreceptors—sensitive to short (S), medium (M), or long (L) wavelengths of light—and transformed into the perception of color. A straightforward mapping of the S-, M-, and L-cone activations to perceived color is complicated by stimulus-independent variations in the cone signals introduced by both extrinsic and intrinsic factors. For example, changes in the wavelength composition of light illuminating a surface can cause considerable variation in the spectrum of light arriving at the retina and consequently in the signals encoded by the trichromatic cone mosaic. Achieving an invariant perception of object color (i.e., color constancy) thus requires the visual system to estimate and discount the spectral properties of the illuminant, a task that humans perform remarkably well under naturalistic conditions (Witzel & Gegenfurtner, 2018).

Even when the characteristics of the external environment are kept constant, additional variability in chromatic encoding can arise from differences in the intrinsic properties of the visual system. These anatomic differences occur both within and between individual observers with ostensibly normal color vision (for a review, see Bosten, 2022). For example, spectral filtering by the macular pigment and senescent changes in crystalline lens transmission modify the spectral distribution of light before it arrives at the retina, causing spectral sensitivity to vary with retinal locus and observer age (Hammond, Wooten, & Snodderly, 1997; Hammond & Caruso–Avery, 2000; Pokorny, Smith, & Lutze, 1987; Sharpe, Stockman, Knau, & Jägle, 1998). Likewise, individual differences in the spectral tuning and relative numerosity of the L- and M-cones mean that the signals available to post-receptoral chromatic mechanisms will vary from one observer to the next (Carroll, Neitz, & Neitz, 2002; Hofer, Carroll, Neitz, Neitz, & Williams, 2005; Neitz & Neitz, 2011).

Despite these sources of variability, color appearance is largely consistent within and across observers. Color appearance between the fovea and periphery is much more similar than predicted by changes in spectral sensitivity due to macular pigment density (Beer, Wortman, Horwitz, & MacLeod, 2005; Webster, Halen, Meyers, Winkler, & Werner, 2010), and color vision is relatively stable across the life span despite changes in lens density (Kraft & Werner, 1999; Schefrin & Werner, 1993; Werner & Schefrin, 1993). Furthermore, observers tend to agree on the color of objects more consistently than variations in their spectral sensitivities would suggest, indicating the presence of compensatory mechanisms that provide an approximate perceptual constancy (Emery & Webster, 2019). These processes presumably exist because the goal of the visual system is not to provide an exact reconstruction of the retinal image but rather to use retinal signals to make inferences about the properties of the external world, including the color of objects. Realizing this goal completely would imply that the appearance of a stimulus of a given spectral composition should be consistent between observers and independent of its size, duration, and location in the visual field. In this study, we examine the extent to which such perceptual invariance is upheld for unique yellow, one of the four phenomenologically pure “unique” hues (along with red, green, and blue) historically thought to represent the equilibrium points of the color-opponent mechanisms (Hurvich & Jameson, 1957; Larimer, Krantz, & Cicerone, 1974).

Of the unique hues, yellow has garnered heightened interest because observers are generally consistent in selecting the wavelength that they perceive to be uniquely yellow, typically in the range of 570 to 580 nm (Abramov & Gordon, 2005; Schefrin & Werner, 1990; Webster, Miyahara, Malkoc, & Raker, 2000). Previous studies have shown that unique yellow is robust to changes in certain test parameters, including stimulus luminance (Ayama, Nakatsue, & Kaiser, 1987; Larimer et al., 1974; Schefrin & Werner, 1990; but see Savoie, 1973), saturation (Burns, Elsner, Pokorny, & Smith, 1984), spectral bandwidth (Mizokami, Werner, Crognale, & Webster, 2006), presentation duration (Abramov & Gordon, 2005), and size (Abramov & Gordon, 2005; Nerger, Volbrecht, & Ayde, 1995), as well as retinal eccentricity (Nerger et al., 1995; Parry, McKeefry, & Murray, 2006). Unique yellow is also independent of individual differences in retinal anatomy: The ratio of L- to M-cones in the retina can range from 0.4 to 13 in color-normal individuals (Carroll et al., 2002; Hofer et al., 2005), but this variation in spectral demographics does not appear to have any meaningful impact on the wavelength perceived as uniquely yellow for large-field stimuli (Mollon & Jordan, 1997; Neitz, Carroll, Yamauchi, Neitz, & Williams, 2002; Yamauchi et al., 2002). Furthermore, prolonged exposure to spectrally altered environments can induce shifts in unique yellow (Belmore & Shevell, 2008; Neitz et al., 2002; Welbourne, Morland, & Wade, 2015), suggesting that the L- and M-cone inputs to higher-order mechanisms responsible for perceiving pure yellow are normalized according to the average spectral diet of the mechanisms (Mollon, 2006; Pokorny & Smith, 1977).

Although the spectral locus of unique yellow remains anchored across a broad range of stimulus manipulations, previous work suggests this invariance may begin to waver when the retinal profile of the stimulus becomes restricted in space and/or time. Otake and Cicerone (2000) found that unique yellow shifted to increasingly longer wavelengths as their briefly presented (200 ms) monochromatic test flashes were reduced in diameter from 10 to 3 arcmin. Their results were interpreted using a neurobiological model in which pure yellow sensations depend on the retinal L/M-cone ratio, with the red–green equilibrium point shifting to longer wavelengths as the relative number of L-cones sampling the stimulus decreases. Similarly, Nagy (1979) observed that unique yellow shifted toward longer wavelengths as a 0.6° test flash was reduced in duration from 1,000 ms to 17 ms, although the effect was dependent on stimulus intensity and did not appear to manifest at lower light levels (Abramov & Gordon, 2005).

Elucidating the conditions under which the invariance of unique yellow no longer holds may provide insights into the properties of the mechanism(s) involved in otherwise maintaining its stability. However, the studies described above all feature three potentially important sources of uncertainty. First, ocular aberrations, which degrade fine details in the retinal image, vary between subjects and are difficult to monitor and correct with conventional optics. Second, incessant and unpredictable fixational eye movements introduce variability in the position of the stimulus on the retina from one trial to the next. Third, the precise spectral topography of an observer's cone mosaic is generally not known. Collectively, these factors preclude a secure understanding of how wavelength information is represented in the earliest stages of the visual system and thus obscure how downstream chromatic mechanisms are being driven, particularly when test flashes are brief and small.

In the present study, we used an adaptive optics scanning laser ophthalmoscope (AOSLO) to correct both low- and high-order aberrations and monitor the retinal position of the stimulus with cellular precision to assess how foveal measurements of unique yellow depend on the size and duration of diffraction-limited stimuli. In a subset of subjects whose parafoveal cone mosaics have been spectrally classified via optoretinography, we also examined the relationship between local spectral demographics and the wavelength of unique yellow as spot size is decreased.

## Methods

### Subjects

**Figure 1.**
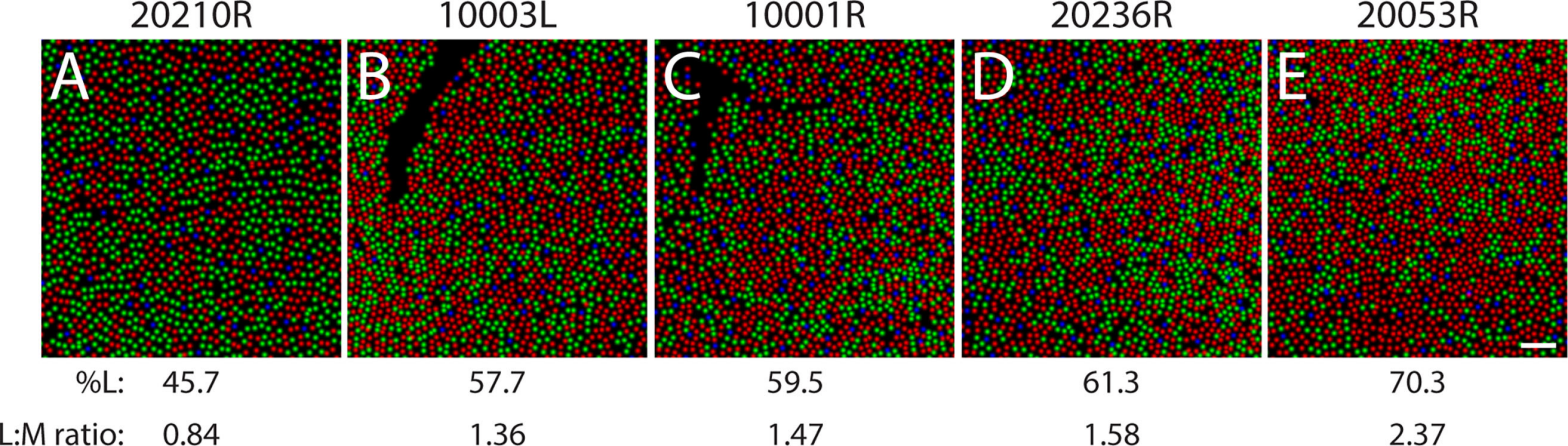
Retinal images at locations where cones have been classified with AO-OCT. For illustrative purposes, cone spectral identity is represented by color (L-, M-, and S-cones are red, green, and blue, respectively). Retinal eccentricities: (**A**) 20210R: 2.0° temporal; (**B**) 10003L: 1.7° temporal; (**C**) 10001R at 2.0° temporal; (**D**) 20236: 1.5° temporal; (**E**) 20053R: 1.5° inferior. Scale bar in (**E**) indicates 5 arcmin of visual angle.

The subject pool for the experiments described below consisted of 10 color-normal subjects, confirmed via testing with a Neitz anomaloscope or by a pseudoisochromatic plate test administered under standard illuminant C. Not all subjects participated in every experiment. Table 1 contains information about each subject. Unique yellow was measured at the fovea using a staircase procedure in eight subjects; four of these subjects (10001R, 20075L, 20206R, and 20210L) also participated in the foveal hue scaling experiment. In the parafovea (∼1.5° to 2° eccentricity), unique yellow was determined for four subjects (10001R, 10003L, 20053R, and 20236R) whose parafoveal cone mosaics were spectrally classified via adaptive optics optical coherence tomography (AO-OCT)–based optoretinography (Pandiyan et al., 2020; Pandiyan et al., 2022). Subject 20210L, who participated in the foveal measurements, had also been classified using optoretinography but was unavailable for parafoveal unique yellow measurements. The spectrally classified mosaics of these subjects are shown in Figure 1. Prior to participating in the study, subjects gave informed written consent. All procedures adhered to the tenets of the Declaration of Helsinki and were approved by local authorities at the University of California, Berkeley and the University of Washington.

**Table 1.** Subject characteristics, level of participation (eccentricities tested, hue scaling), and cone classification status. Notes: F = female; M = male; N = no; Y = yes.

				Test eccentricity			
Subject ID	Age, Y	Sex	Ethnicity	Fovea	Parafovea	Hue scaling	Classified cone mosaic
10001R	37	M	European	Y	Y	Y	Y
10003L	55	M	European	N	Y	N	Y
20053R	39	M	South Asian	N	Y	N	Y
20075L	32	F	European	Y	N	Y	N
20206R	36	M	European	Y	N	Y	N
20210L	33	M	European	Y	N	Y	Y^†^
20212L	26	M	European	Y	N	N	N
20214R	26	F	European/East Asian	Y	N	N	N
20230R	27	F	European	Y	N	N	N
20236R	41	M	South Asian	Y	Y	N	Y

† ORG classification was done in the fellow eye for a separate study.

### Equipment

A multiwavelength AOSLO was used for simultaneous retinal imaging and stimulus generation. The AOSLO has been described in detail (Harmening, Tuten, Roorda, & Sincich, 2014; Roorda et al., 2002; Wang et al., 2019). In the current setup, four wavelengths (λ = 940 nm, 840 nm, 680 nm, and 543 nm) were selected from a supercontinuum light source (SuperK EXTREME; NKT Photonics, Birkerød, Denmark). A custom-built Shack-Hartmann wavefront sensor (λ = 940 nm; average power at the cornea: 59 µW) was used to measure the optical aberrations of the eye with closed-loop correction via a deformable mirror (DM97-08; ALPAO, Montbonnot-Saint-Martin, France). Infrared light (λ = 840 nm; average power at the cornea: 116 µW) was used for confocal retinal imaging, with light reflected back from the photoreceptor layers captured by a photo-multiplier tube (photosensor module H7422; Hamamatsu Photonics, Hamamatsu, Japan) placed behind a pinhole aperture subtending 0.43 Airy disk diameters. Two wavelengths in the green and red regions of the visible spectrum (λ = 543 ± 13.5 nm and 680 ± 14.9 nm) were used for stimulus generation. The field of view for the AOSLO system was 0.91°, corresponding to a sampling resolution of approximately 0.11 arcmin per pixel.

### Imaging and stimulation with AOSLO

Subjects were dilated and cycloplegia was induced with 1% tropicamide and 2.5% phenylephrine ophthalmic solutions 15 minutes prior to the experiment. A bite bar attached to an X/Y/Z translation stage was used to align the subject's eye to the pupil plane of the AOSLO system. Image quality was monitored throughout each experiment session, with the AOSLO correcting aberrations in a closed loop over a 6- to 7-mm pupil diameter.

The AOSLO scanned light onto the subject's retina in a raster pattern using two scanning mirrors: a galvanometer operating vertically at ∼30 Hz and a faster, resonant scanner operating at 16 kHz oriented horizontally. The frame rate for both imaging and stimulus presentation is dependent on the scanning frequency of the galvanometer. Stimuli were generated by modulating the intensities of the “green” (543 nm) and “red” (680 nm) primaries at the appropriate points in the raster scan using two independent, fiber-coupled acousto-optic modulators (AOMs; Brimrose Corp, Sparks Glencoe, MD, USA). Each stimulus pixel was rendered in a discrete time interval spanning 50 ns; hence, the exact temporal characteristics of the stimulus depended on both the scanning dynamics and the number of pixels it contained. For these reasons, we have opted to specify stimulus durations in number of frames rather than absolute units of time (see below).

The floor of the output range from the AOM is limited by a nonzero extinction ratio. The background light from the AOSLO was composed of leaks from the two primary channels (combined leaks: 3.19 log Trolands), in addition to dimly visible light from the 840-nm imaging channel (3.24 cd/m^2^). Additional background light was provided by an RGB projector (DLP LightCrafter 4500; Texas Instruments, Dallas, TX, USA), configured in Maxwellian view and calibrated using a spectroradiometer (SpectraScan PR650; Photo Research Inc., Chatsworth, CA, USA). The subject aligned a small square patch, approximately metameric to equal energy white (MacLeod–Boynton coordinates r = 0.7078, b = 0.0192), to be spatially coincident with the AOSLO raster. The raster-aligned patch was surrounded by a larger rectangular achromatic background 18° in diameter. The luminances of the patch and larger background generated by the projector were 4.47 log Trolands and 4.17 log Trolands, respectively. The most recent projector calibration revealed that the relative powers of the LED primaries had drifted from their presumed values, such that the MacLeod–Boynton chromaticity coordinates of the projector were blue-shifted (r = 0.6913, b = 0.0365) for some of our unique yellow measurements. We note that this is still approximately within the range of chromaticities that corresponds to absolute white for color-normal observers (Bosten, Beer, & MacLeod, 2015).

During foveal imaging, the subject was instructed to fixate as well as possible on the center of the projector-generated white square patch, which coincided with the center of the imaging raster. During parafoveal imaging, the subject fixated on a small black spot presented through the projector and placed at the appropriate distance from the imaging raster. Stimuli always appeared at the center of the imaging raster. Thus, the exact location of the stimulus on the retina was influenced by the subject's fixational eye movements and naturally varied between trials.

**Figure 2.**
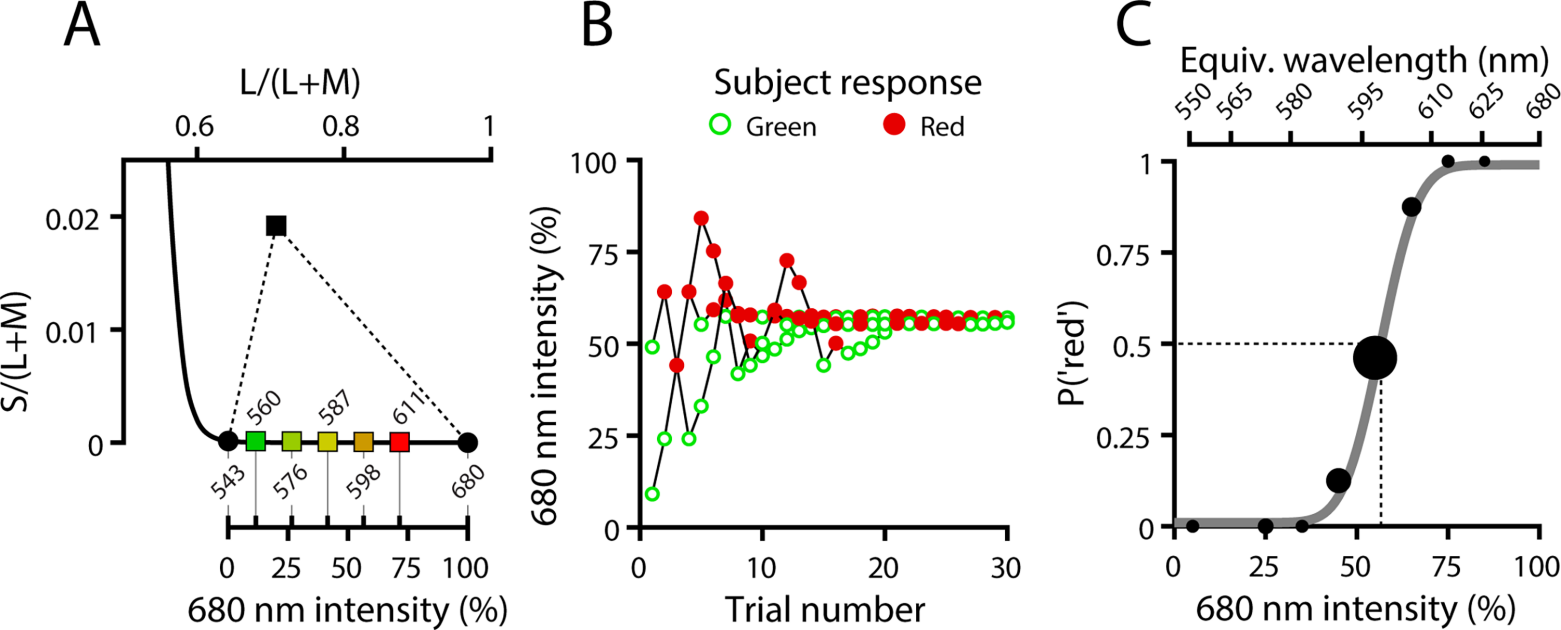
(**A**) Stimulus gamut in MacLeod–Boynton chromaticity space, defined by the achromatic projector background (black square) and AOSLO primaries (black circles). The stimuli used for hue scaling are shown as colored squares along the spectral locus, with dominant wavelengths (in nm) for each stimulus labeled adjacent to the marker. (**B**) Single-session adaptive staircase data for 20212L, for the 3.2 arcmin, one-frame foveal test condition. Trial markers are color-coded by subject response (red or green). (**C**) Psychometric function fitted from the staircase data in (**B**). Black circles indicate the proportion of trials the subject responded red; marker area is scaled to the number of trials for each intensity. Black dashed lines denote the 680-nm primary intensity that produced a subjective red–green equilibrium (i.e., “unique yellow”).

The two AOSLO primaries (543 nm and 680 nm) were used to generate stimuli metameric to intermediate spectral wavelengths (Figure 2). The maximum outputs of the two channels were measured using a power meter (PM100D; Thorlabs Inc., Newton, NJ, USA) with neutral density (ND) filters placed at the source so that the red and green primaries were in approximate equiluminance, according to the standard observer luminosity function (Sharpe, Stockman, Jagla, & Jägle, 2005). To account for small residual differences in luminosity that could not be eliminated with ND filters, in addition to slight day-to-day power fluctuations of the AOSLO light source, the maximum output of the more luminous channel was scaled down to match that of the less luminous channel. Thus, a value of 1 on the arbitrary unit (au) scale used here represents the maximum intensity from each channel used during the experiment after equating luminosity, where 1 au = 5 log Trolands. The floor (0 au) represents the total background light, including contributions from the AOSLO and projector. Stimuli were specified by the proportion of the total stimulus luminance contributed by the red (i.e., 680 nm) primary and converted to equivalent wavelength where necessary.

### Correction of chromatic aberration

To ensure the 543-nm and 680-nm AOSLO primaries were optimally focused and spatially coincident on the photoreceptor layer during testing, we measured and corrected the longitudinal and transverse chromatic aberrations (LCA and TCA, respectively) that arise from chromatic dispersion in the eye and in the instrument. LCA was corrected by adjusting the relative vergences of the sources at their entry points in the light delivery arm of the AOSLO, using 680 nm as the reference wavelength, following the expected difference in refraction from published LCA models (Atchison & Smith, 2005). Because individual LCA can vary from these normative values, we began each session by collecting full-field images of the cone mosaic using the 543-nm and 680-nm channels while system focus was adjusted; all subsequent testing was done at a focus setting that mutually optimized image quality in the two stimulation channels.

As described below, TCA between the two stimulus wavelengths was corrected either using an image-based or subjective procedure. Pupil tracking was used to minimize changes in TCA that occur with pupil displacements (Boehm, Privitera, Schmidt, & Roorda, 2019; Domdei, Linden, Reiniger, Holz, & Harmening, 2019). Back-illuminated infrared light from the retina was used to gather images of the pupil, and the first Purkinje image was used to track changes in pupil position relative to a reference location that was determined by the experimenter at the beginning of each experiment session to optimize the quality of retinal images obtained with the AOSLO system.

For parafoveal testing, an image-based procedure (Harmening, Tiruveedhula, Roorda, & Sincich, 2012) was used to measure the transverse chromatic offsets (TCOs) between the 543-nm and 680-nm primaries (relative to the 840-nm imaging light) that arise from a combination of the eye's TCA and any residual misalignment of the AOSLO channels. Knowledge of these offsets is essential for maintaining spatial alignment between the red and green primaries and determining where the stimuli landed on the cone mosaic. In the central fovea, where cones are densely packed and hard to resolve, the low signal to noise of the 543-nm images precluded reliable image-based estimates of TCO. In this case, foveal TCOs were instead measured using a psychophysical procedure in which stimuli rendered by the 543-nm and 680-nm primaries were aligned. For some subjects, the subjective alignment procedure employed a stimulus similar to one that was previously shown to produce good agreement between subjective and image-based TCO estimates (shown in Figure 6 of Domdei et al., 2019). Some subjects preferred a simpler procedure in which the relative positions of middle- and long-wavelength components of 10.7-by-10.7 arcmin square were adjusted until it appeared uniform in color. During the experiment, subjects were instructed to monitor for misalignments of the stimulus components, which would manifest as salient red or green fringes at the stimulus edges. These instances were infrequent and typically corresponded with pupil drift. Whenever noted, subjects repeated the TCO measurement procedure described above before resuming data collection.

### Staircase measurements of unique yellow

We used an adaptive staircase procedure to determine unique yellow as a function of stimulus size and duration. The subject's task was to report whether the stimulus appeared red (redder, reddish) or green (greener, greenish) in a two-alternative forced-response paradigm. The relative intensities of the red and green primaries converged to values such that the subject had a 50% probability of responding “redder” or “greener”—which occurred when the subject perceived the stimulus to be uniquely yellow.

Stimuli were squares with side lengths of 1.1, 3.2, or 10.7 arcminutes when presented foveally and 3.2, 7.5, or 10.7 arcminutes when presented parafoveally. For one subject (20236R), a 7.0-arcmin stimulus was inadvertently used instead of 7.5 in the parafovea. Stimuli were on for 1, 4, or 15 AOSLO frames (which correspond to nominal durations^1^ of <10, 100, or 467 ms; see footnote below). Nine of the 10 subjects completed a follow-up experiment where unique yellow was measured for stimuli composed of the full AOSLO raster (0.9° × 0.9°). This was to provide a comparison with unique yellow estimates from previous studies, which typically use stimuli on the order of 1° or larger.

The subject initiated each trial by key press, which triggered a 1-second interval during which the stimulus was presented and a 30-frame video of the retina was recorded. The location of the stimulus on the retina was encoded into the video using a digital marker that could be identified in subsequent analyses. After viewing the stimulus, the subject logged their response (“redder” or “greener”) by pressing the corresponding key on a gamepad or keyboard. If the stimulus appeared to be uniquely yellow (i.e., having no hint of either red or green), participants were instructed to respond randomly and avoid biasing their responses. If the subject blinked or made an eye movement during stimulus delivery, they were given the option to repeat the presentation, although this option was rarely used.

Within a single experimental session, subjects completed blocks of 180–240 trials in which at least three stimulus conditions (distinct combinations of the three stimulus sizes and three stimulus durations described above) were tested. For each stimulus condition within a measurement block, two independent staircases operated in parallel to control stimulus chromaticity. The staircases were guided by an adaptive, 1-up/1-down algorithm, which controlled the intensity value of the red component of the stimulus, with the green intensity level simultaneously adjusted so that the luminance of the mixture remained constant. The stimulus condition and staircase index were randomly selected on each trial so that the subject could not predict the spatial, temporal, or chromatic characteristics of the stimulus based on the preceding trial. A typical experimental session contained three measurement blocks, corresponding to ∼600 trials, with a minimum of 40 trials completed for each stimulus condition. Full-raster measurements were collected in separate measurement blocks of 40 trials, comprising two randomly interleaved staircases.

For each stimulus condition, a single psychometric function was fitted to data compiled from all staircases obtained in the experimental session. This function relates the probability of a “red” response to the relative intensity of the red (680 nm) component of the stimulus (Figure 2C). Unique yellow occurs when the probability of responding “red” is 50% (i.e., at “red–green equilibrium”). The abscissa of the 50% point gives the relative intensity of the red (680 nm) primary required for unique yellow. This value (along with the green primary intensity and the cone fundamentals) can be used to locate the stimulus in MacLeod–Boynton space and thereby convert from red intensity to dominant wavelength (Figure 2).

For the foveal unique yellow measurements, all subjects except for 20075L and 20210L participated in multiple experimental sessions over different days. For parafoveal measurements, only 10001R participated in more than one session. Each experimental session featured a similar allocation of trials per stimulus condition. A “fit-then-pool” approach was taken with data collected over multiple days: Psychometric curves were fit to each daily data set, yielding separate unique yellow estimates. These estimates were then averaged to produce a final unique yellow value for the given condition. In all analyses, the rare out-of-gamut unique yellow estimates (i.e., less than 543 nm or above 680 nm), as well as estimates for which the bootstrapped confidence intervals were undefined, were excluded as outliers.

### Hue scaling

In the present study, unique yellow was measured by identifying the mixture of red and green primaries that appeared to contain no trace of redness or greenness. With spatially restricted stimuli sampled by only a few cones, we considered whether it might be possible to arrive at a perceived red/green equilibrium color other than yellow, such as white. To characterize the color appearance of stimuli generated by mixing the AOSLO primaries in various proportions, a subset of subjects performed hue scaling with foveally presented stimuli. Hue-scaling stimuli were small squares, 1.1 arcminutes in diameter, presented for one of three stimulus durations (1, 4, or 15 frames). A fourth stimulus condition, where the stimulus was 10.7 arcminutes in diameter and presented for 15 frames, was also included. Five, equally spaced stimulus levels (i.e., percent red) were used, where the midpoint was 41.5% red (based on an average unique yellow estimate for these stimulus conditions in a preliminary experiment). The other stimulus levels were one and two steps of 15% red away from the midpoint in either direction, spanning 60% of the available range of stimuli achievable by mixture of the two wavelengths. The MacLeod–Boynton chromaticities and equivalent wavelengths of the hue scaling stimuli are shown in Figure 2A.

All trials were self-paced: The subject initiated the trial with a button press on a gamepad controller. After viewing the stimulus, the subject indicated, with 10 button presses, the proportion of the stimulus that was seen as red, green, blue, yellow, or white. Each button press represented 10% of the total chromatic content of the stimulus. Each stimulus condition/level was presented to the subject 15 times over the course of three blocks of trials where the various stimulus conditions were randomly interleaved. For each stimulus condition, mean hue ratings were calculated over the range of tested chromaticities and plotted in uniform appearance diagrams (UADs) as polar coordinates [saturation (ρ), hue angle (θ)], where

$$\begin{array}{ccc} \;\rho \; & = & 100-\%\;\mathrm{white},\;\mathrm{and}\\ \;\theta \; & = & \mathrm{atan}2\left(\%\;\mathrm{yellow}-\%\;\mathrm{blue},\;\%\mathrm{red}-\%\;\mathrm{green}\right)\;\mathrm{mod}\;2\pi . \end{array}$$

### Analysis of stimulus light delivery and cone spectral topography

We examined the impact of local cone spectral topography on unique yellow for each of the stimulus conditions presented in the parafovea. This required knowledge of where the stimulus landed on the retina on each trial, as well as an approximation of the retinal image produced by the stimulus. Videos were grouped by condition and session, and a retina-based simultaneous localization and mapping software package (R-SLAM; Shenoy, Fong, Tan, Roorda, & Ng, 2021) was used to generate high signal-to-noise, spatially registered images from sets of videos, referred to as “retinal maps” from here on. Stimulus locations, extracted by locating the digital crosses inserted in the raw video frames to mark the retinal locus of stimulus delivery, were then registered to R-SLAM–generated retinal maps so that stimulus deliveries for all trials could be plotted in a common coordinate system for each subject. Additionally, cone spectral identity labels were transferred from AO-OCT images to the R-SLAM–generated retinal maps using custom software (Pandiyan et al., 2022).

To calculate the L/M ratio of the retinal region illuminated by the stimulus, we began by computing an approximation of the stimulus light distribution on the retina at the recorded delivery location, for each frame of each video. The retinal image of the stimulus was approximated by convolving, in turn, the red and green components of the square stimulus by the appropriate (i.e., wavelength-specific) point spread function (PSF), computed for a diffraction-limited light delivery system with a 6.5-mm pupil and then aberrated by 0.05 D of defocus according to previous estimates of residual blur in our apparatus (Harmening et al., 2014). The red and green stimulus components, blurred by diffraction and a small amount of defocus, were then weighted by the respective red and green stimulus intensities and recombined. Next, cones in the retinal image, labeled as L, M, or S, were modeled as circles with diameters approximately equal to the full width at half maximum of the cone aperture function for the test eccentricity (∼0.6 arcmin; Curcio, Sloan, Kalina, & Hendrickson, 1990). Finally, the arrays of L- and M-cones in this image were multiplied (separately) by the PSF-convolved stimulus. The product of the L-cone array with the stimulus was integrated to approximate the number of L-cones stimulated and likewise for M-cones. The ratio of these values is the L/M ratio for the given frame. On multiframe trials, the trial L/M ratio was taken as the average of the L/M ratios for the individual stimulation frames and thus will incorporate the effects of retinal motion occurring between frames.

To compute the spatial distribution of delivered light, the PSF-convolved red component of the stimulus was scaled by the relative intensity (converted to luminance as described above) of the red primary and summed across trials. The same calculation was performed for the green stimulus, and the summed red and green luminance distributions were averaged. This average was used to compute contours which encircle 50% or 90% of the total luminous power delivered across trials for each condition and session.

### Linear mixed-effects model

To quantify the effects of size and duration on unique yellow, we fit our data to the following linear mixed-effects model (LMM):

$$\begin{array}{ccc} & & \;y_{\mathrm{ijk}}=\beta_{0}+b_{0i}+\left(\beta_{1}+b_{1i}\right)\;\mathrm{diamete}r_{j}\\ & & \;+\left(\beta_{2}+b_{2i}\right)\;\mathrm{duratio}n_{j}\\ & & \;+\left(\beta_{3}+b_{3i}\right)\left(\mathrm{diamete}r_{j}\times \mathrm{duratio}n_{j}\right)+{ɛ}_{\mathrm{ijk}} \end{array}$$

where β_n_ is the nth fixed-effect coefficient, b_mi_ is the mth random-effect coefficient for the ith subject, and ε_ijk_ is the error term for the kth measurement of the jth condition in the ith subject.

We chose an LMM because our data can be clustered by subject, which violates the independence assumption of an analysis of variance (ANOVA), and because our repeated measures are unbalanced between subjects, which precludes the use of a repeated-measures ANOVA.

The primary purpose of our model is to estimate, across observers, the magnitude and direction of size's effect on unique yellow (β_1_) and the magnitude and direction of duration's effect on unique yellow (β_2_). Additionally, we include a term that estimates the wavelength unique yellow approaches as stimuli are made infinitesimally small and brief (β_0_), as well as a term that accounts for interactions between size and duration (β_3_). To account for individual differences in the locus of unique yellow for static stimulus parameters and individual differences in the strength of the effects of size and duration, we included subject-specific random effects for each fixed-effect regressor.

We fit our model using the fitlme function in MATLAB, employing the restricted maximum likelihood method. All statistical analyses were run in MATLAB R2021a.

## Results

### Variability of unique yellow in the fovea

**Figure 3.**
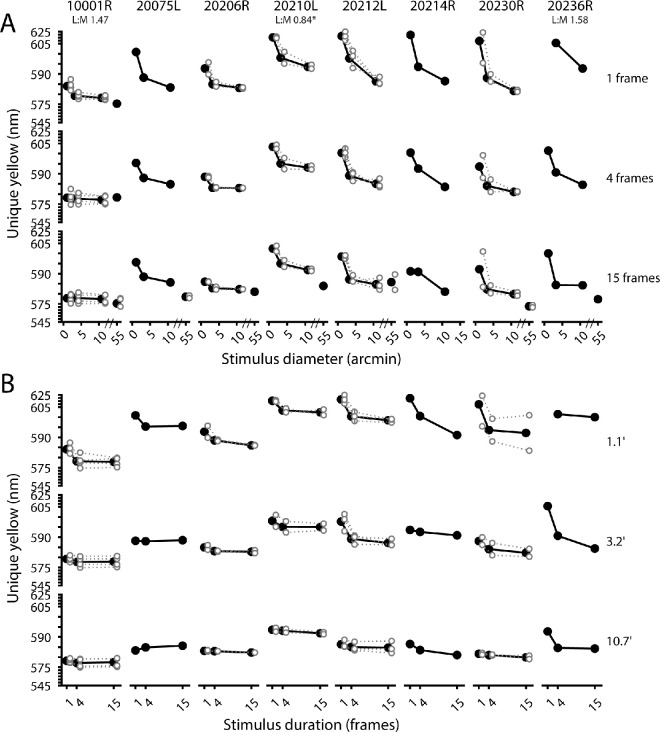
Foveal measurements of unique yellow as a function of stimulus size and duration. (**A**) Unique yellow-versus-diameter functions for eight color-normal subjects. Data are arranged in rows according to the stimulus duration in AOSLO frames (frame rate: 30 Hz). The 1-, 4-, and 15-frame conditions are nominally equivalent to <10-, 100-, and 467-ms stimulus durations, respectively. Filled circles indicate mean unique yellow settings; open circles indicate individual measurements for subjects who participated in more than one experimental session. (**B**) Data from (**A**) are replotted to illustrate the dependence of unique yellow on stimulus duration for stimuli of various sizes. Note: for both panels, the y-axis is compressed at the extreme ends of the scale for visualization purposes, and the 1.1 arcmin, one-frame data point for subject 20236R (rightmost column) was excluded due to a poor psychometric function fit to the staircase data. For participants with spectrally classified cone mosaics, parafoveal L/M ratios are listed beneath the subject identifier. *ORG classification in subject 20210L was done in the fellow eye as part of another study, as noted in Table 1. The data from Figure 3 are shown as a color-coded matrix in Supplementary Figure S1.

Red–green equilibrium measurements were made for all combinations of stimulus size (1.1, 3.2, or 10.7 arcmin) and duration (1, 4, or 15 frames) in the central fovea. In addition, full-raster (55 × 55 arcmin) estimates of unique yellow were obtained in seven subjects for the 15-frame stimulus duration. In Figure 3A, unique yellow estimates are shown as a function of stimulus diameter for all eight subjects. In Figure 3B, unique yellow measurements are plotted as a function of stimulus duration. Most subjects showed a noticeable shift in unique yellow to longer wavelengths as either stimulus size or duration is reduced. This implies that smaller, more briefly presented stimuli tended to appear greener than larger, longer-duration stimuli. The greatest shift occurred between the small (1.1 arcmin) and medium (3.2 arcmin) stimulus sizes, for the one-frame condition. This change in unique yellow between the small- and medium-sized stimuli appears to be mitigated by increased stimulus duration. Several subjects showed a drop in the wavelength of unique yellow between 10.7 arcmin and 55 arcmin, suggesting the unique yellow versus diameter functions for certain observers may not reach an asymptote until the stimulus size is increased beyond 10 arcmin. Across subjects, the mean foveal unique yellow measurement for the 55 arcmin stimulus was 578.4 ± 3.0 nm (mean ± *SEM*).

**Figure 4.**
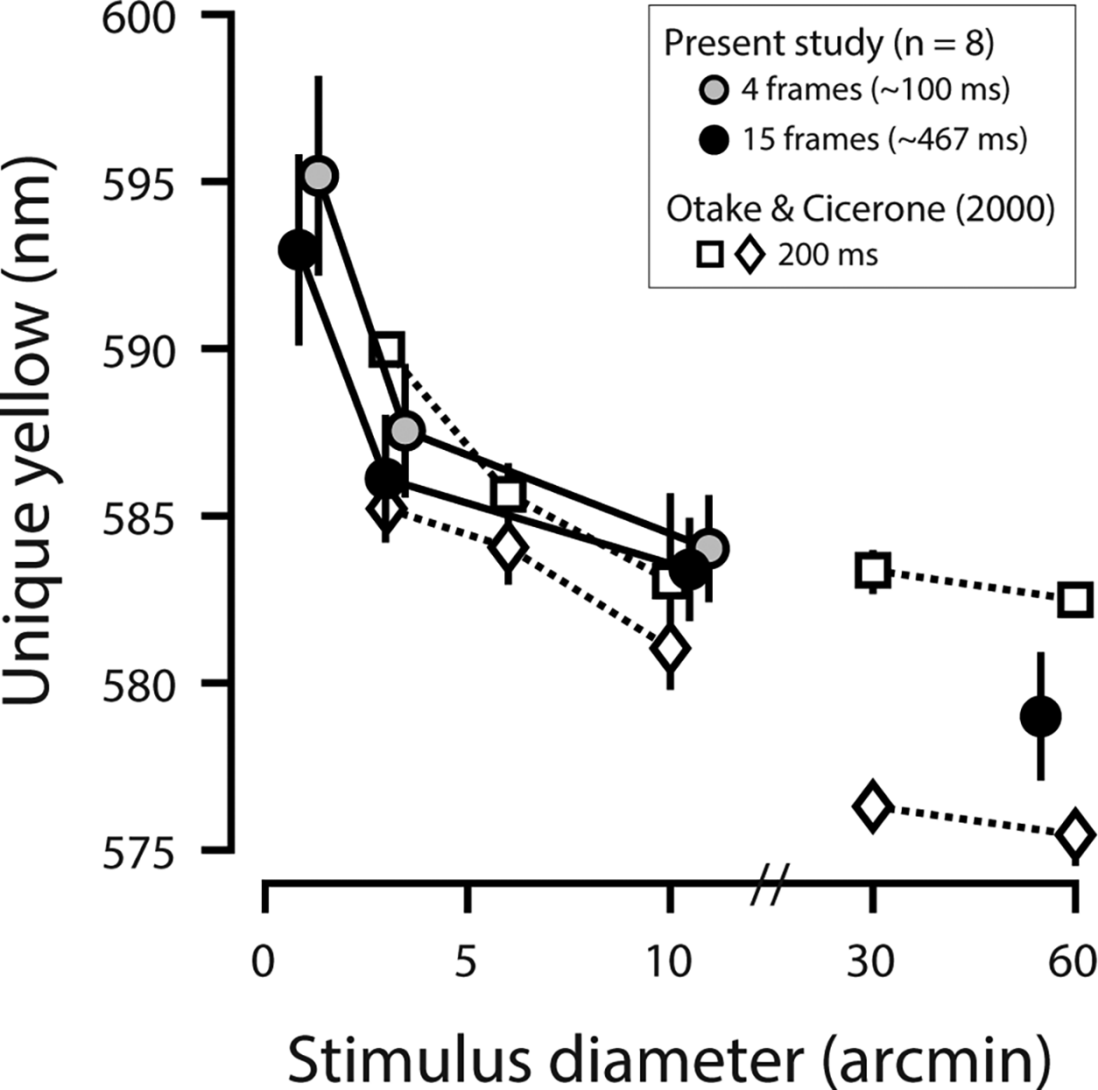
Unique yellow versus stimulus size. Gray markers plot the foveal unique yellow measurements as a function of stimulus size for the four-frame condition (nominally 100 ms) (Figure 3A, middle row), averaged across eight subjects. Unique yellow estimates for the 15-frame condition (nominally 467 ms) are shown by the black markers. Error bars are ±1 *SEM*. Gray and black markers are offset horizontally to enhance visualization. Open markers show unique yellow measurements obtained with an intermediate stimulus duration (200 ms) for two subjects, MS (squares) and VC (diamonds), as reported in Otake and Cicerone (2000).

Figure 4 compares our foveal unique yellow measurements to those made by Otake and Cicerone (2000) under comparable conditions. Data from the present study are shown for the four-frame and 15-frame stimuli, whose nominal stimulus durations (100 ms and 467 ms, respectively) bracket the 200-ms presentation time used by Otake and Cicerone (2000). Despite differing methodologies, our foveal unique yellow measurements agree reasonably well with those of Otake and Cicerone.

We fit our data to the linear mixed-effects model described in the Methods. The fixed effects of stimulus diameter (β_1_ = −1.578, *p* = −3.109e-06) and duration (β_2_ = −0*.*851, *p* = 2.635e-06) were significant. Additionally, the fixed interaction between size and duration was significant (β_3_ = 0.094, *p* = 1.069e-05). Therefore, our data confirm the results of Otake and Cicerone that unique yellow depends on the spatiotemporal profile of small, brief flashes.

To assess whether there are meaningful differences in the effects of size and duration between subjects, we performed a likelihood ratio (LR) test on the full model described above and a reduced model in which the only random effect is intercept. Model fits were compared using the Akaike information criterion (AIC). We found that the full model is superior to the reduced model (LR = 61.796, Δ*df* = 9, ΔAIC = 43.8, *p* = 6.0349e-10). Thus, a model that accounts for individual differences in how unique yellow shifts with size and duration better fits our data than a model that assumes no such differences.

### Hue scaling

**Figure 5.**
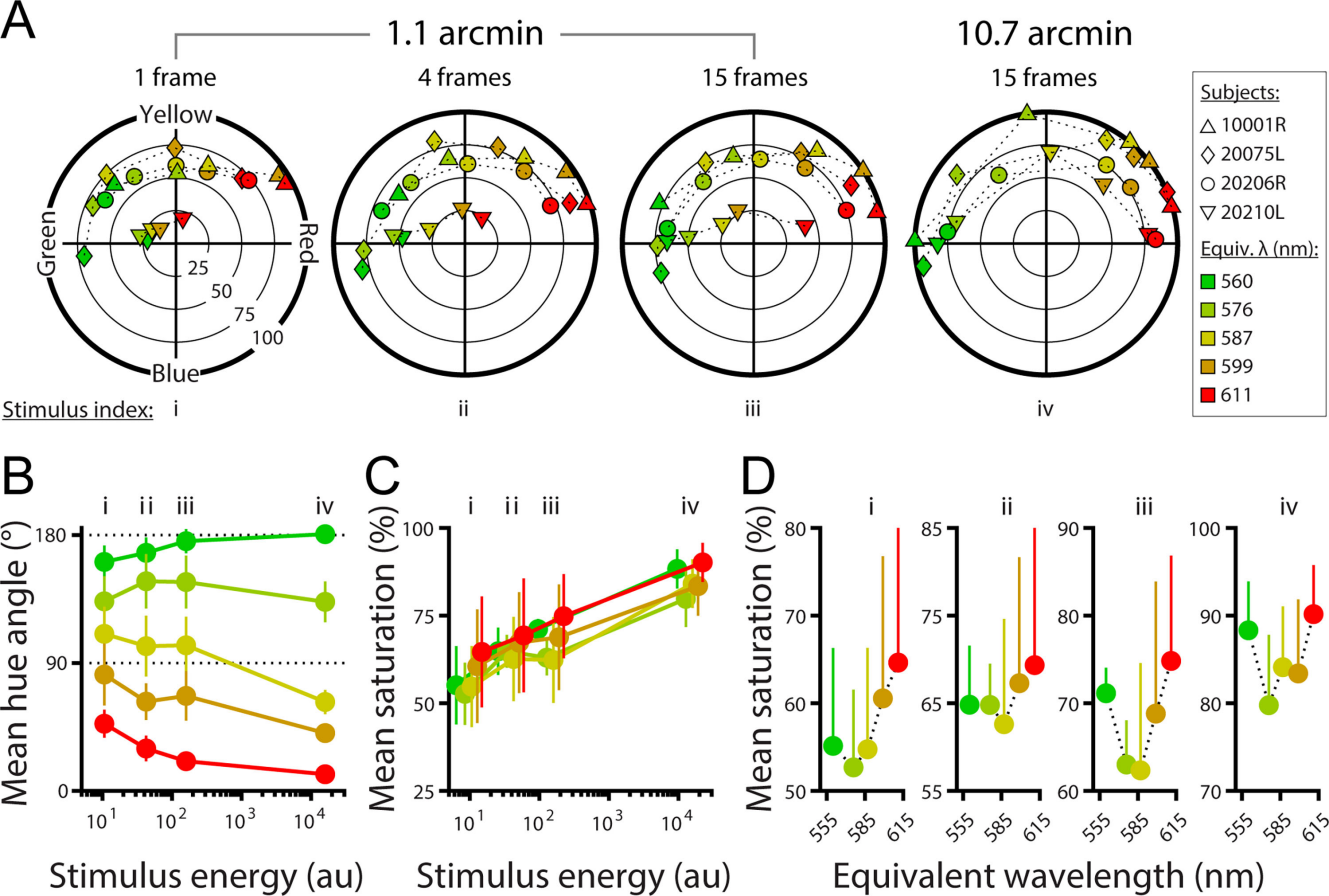
Hue scaling of brief, small-field mixtures of red and green. (**A**) Uniform appearance diagrams for the 1.1 arcmin stimulus at each duration, as well as the 10.7 arcmin stimulus at 15 frames. Different marker shapes indicate different subjects. Markers color indicates stimulus locus in MacLeod–Boynton space, as shown in Figure 2A. Redder markers correspond to stimuli with a higher proportion red (680 nm), and greener markers correspond to stimuli with a higher proportion green (543 nm). (**B**) Mean (± *SEM*) hue angle across subjects versus stimulus energy (diameter × duration), for different mixtures of the red and green primaries. (**C**) Mean (± *SEM*) saturation across subjects versus stimulus energy. (**D**) Mean (± *SEM*) saturation versus stimulus equivalent wavelength, for each of the energy levels indicated by i–iv in (**C**). Note: lower error bars are not plotted in (**D**) for visualization purposes.

Figure 5A shows uniform appearance diagrams derived from the hue-scaling data collected from four subjects. Saturation (100 – %white) is represented as the Euclidean distance from the origin, and hue is represented by angle (θ; measured counterclockwise from the horizontal axis). At the largest size (10.7 arcmin) and duration (15 frames) tested, stimuli with dominant wavelengths between 560 and 611 nm generated highly saturated percepts that ranged in hue between almost pure red (θ = 0°) and pure green (θ = 180°). A reduction in size to 1.1 arcmin caused the stimuli presented for the same duration to appear less saturated and generally more yellow. This effect continued with reductions in stimulus duration from 15 frames to one frame.

**Figure 6.**
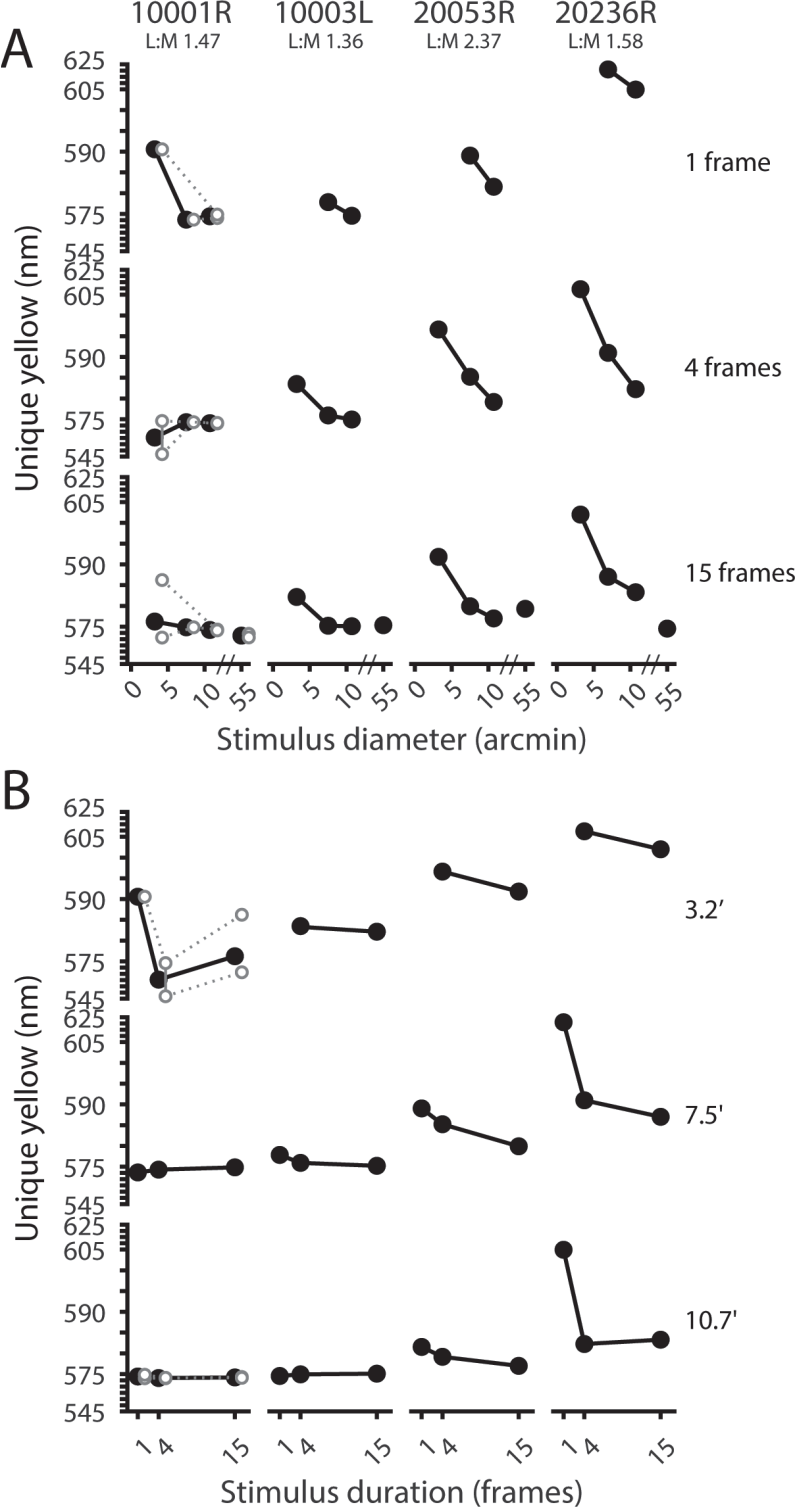
Parafoveal measurements of unique yellow as a function of stimulus size and duration. (**A**) Unique yellow-versus-diameter functions for four color-normal subjects. Data are arranged in rows according to the stimulus duration in AOSLO frames (frame rate: 30 Hz). The 1-, 4-, and 15-frame conditions are nominally equivalent to <10-, 100-, and 467-ms stimulus durations, respectively. Filled circles indicate mean unique yellow settings; open circles indicate individual measurements for subjects who participated in more than one experimental session. Parafoveal L/M ratios are indicated beneath each subject identifier. (**B**) Data from (**A**) are replotted to illustrate the dependence of unique yellow on stimulus duration for stimuli of various sizes. In both panels, the y-axis is compressed at the extreme ends of the scale for visualization purposes. For subjects 10003L, 20053R, and 20236R, data for the 3.2 arcmin, one-frame condition were excluded due to poor psychometric function fits. Note: for subject 20236R, the middle-sized stimulus subtended 7.0 arcmin; these data are plotted with the 7.5 arcmin data obtained from the other participants. The data from Figure 6 are shown as a color-coded matrix in Supplementary Figure S2.

The amount of stimulus light reaching the eye is a function of both size and duration, and these two variables can be collapsed into a single dimension: stimulus energy (stimulus area × duration). Figure 5B shows the general trend of hue angle compression around yellow with reduced stimulus energy (stimulus area × duration). Each set of connected points corresponds to a particular red/green mixture metameric to the wavelengths indicated in Figure 2. The green curve (top) corresponds to the mixture with the least amount of red (680 nm) light, and the consecutive sets below correspond to mixtures with increasing proportions of red light. As stimulus size and duration are reduced, the top two curves in Figure 5B tend to slope downward while the bottom two curves slope upward. This reflects the shift toward yellow of stimuli that appear either reddish or greenish with enough stimulus energy. The middle curve, which represents a mixture metameric to 587 nm (see Figure 2), slopes upward with less stimulus energy. This stimulus appears to contain some redness at the largest size and longest duration and was reported as increasingly greenish as it was made smaller or briefer. This is consistent with the shift in unique yellow to longer wavelengths that occurred when stimuli were smaller and/or more transient, as we observed in the two-alternative forced-response data shown in Figures 3 and 6.

In Figure 5C, saturation is plotted against stimulus energy, demonstrating how apparent saturation increases as stimulus duration or size is increased. When saturation ratings for each combination of stimulus size and duration are plotted against the dominant wavelength of the red–green mixture (Figure 5D), the data suggest that mid-spectrum stimuli were rated as less saturated than stimuli that predominantly contained light from either the red or green primary; this trend appears to be present for all stimulus energy levels we tested. One subject (20210L) tended to rate the smallest stimulus as especially desaturated, compared with other subjects’ ratings. The 611-nm stimulus at 1.1 arcmin and one frame appeared much closer to yellow (θ = 90°) for this subject than for the others. Notably, the unique yellow measurement obtained using the staircase method also exhibited a substantial redshift for the smallest, briefest stimulus (Figure 3). Furthermore, we note that subject 20210L was an exception with a parafoveal L/M ratio below 1, unlike the other spectrally-classified participants in this study whose L/M ratios were greater than 1 (Figure 1).

### Variability of unique yellow in the parafovea

Unique yellow was determined for different stimulus sizes (3.2, 7.5, or 10.7 arcmin) and durations (1, 4, or 15 frames) at about 1.5° from the fovea, targeted to regions of the retina where cone types were known from previous AO-OCT measurements. In Figure 6A, unique yellow is plotted against stimulus size, and in Figure 6B, unique yellow is plotted against stimulus duration, in the exact same manner as in Figure 3. As in the fovea, unique yellow tended to occur at longer wavelengths when stimuli were made smaller or briefer. This effect was more obvious for stimulus size than duration. Interestingly, unique yellow shifted to shorter wavelengths with reduced stimulus size for subject 10001R (at least for the one- and four-frame conditions). As discussed below, this may be due to stimuli falling on an especially L-rich region of this subject's retina, resulting in an overabundance of “red” responses.

We fit the same generalized linear mixed-effects model used for foveal data to our parafoveal data to quantify the effects of stimulus size and duration. The effects of stimulus diameter (β_1_ = *−*2.855, *p* = 0.070) and duration (β_2_ = *−*1.912, *p* = 0*.*082) did not reach a significance threshold of 0.05, nor did the interaction between diameter and duration (β_3_ = 0.180, *p* = 0.085). This is likely because, as mentioned, one of the four subjects (10001R) showed a trend opposite to that of the other subjects (shorter wavelength unique yellow with decreased size). One of the unique yellow estimates for the 3.2 arcmin, four-frame stimulus is especially green-shifted (λ_UY_ = 547.0 nm) for this subject, and the confidence interval for this estimate is quite wide (515.2 to 568.3 nm). The inclusion of random effects of diameter and duration does not significantly improve our model over a reduced model in which diameter and size are solely fixed effects (LR = 12.896, Δ*df* = 9, ΔAIC = 5.1, *p* = 0.16741).

### Unique yellow and the cone mosaic

#### Light delivery contours

**Figure 7.**
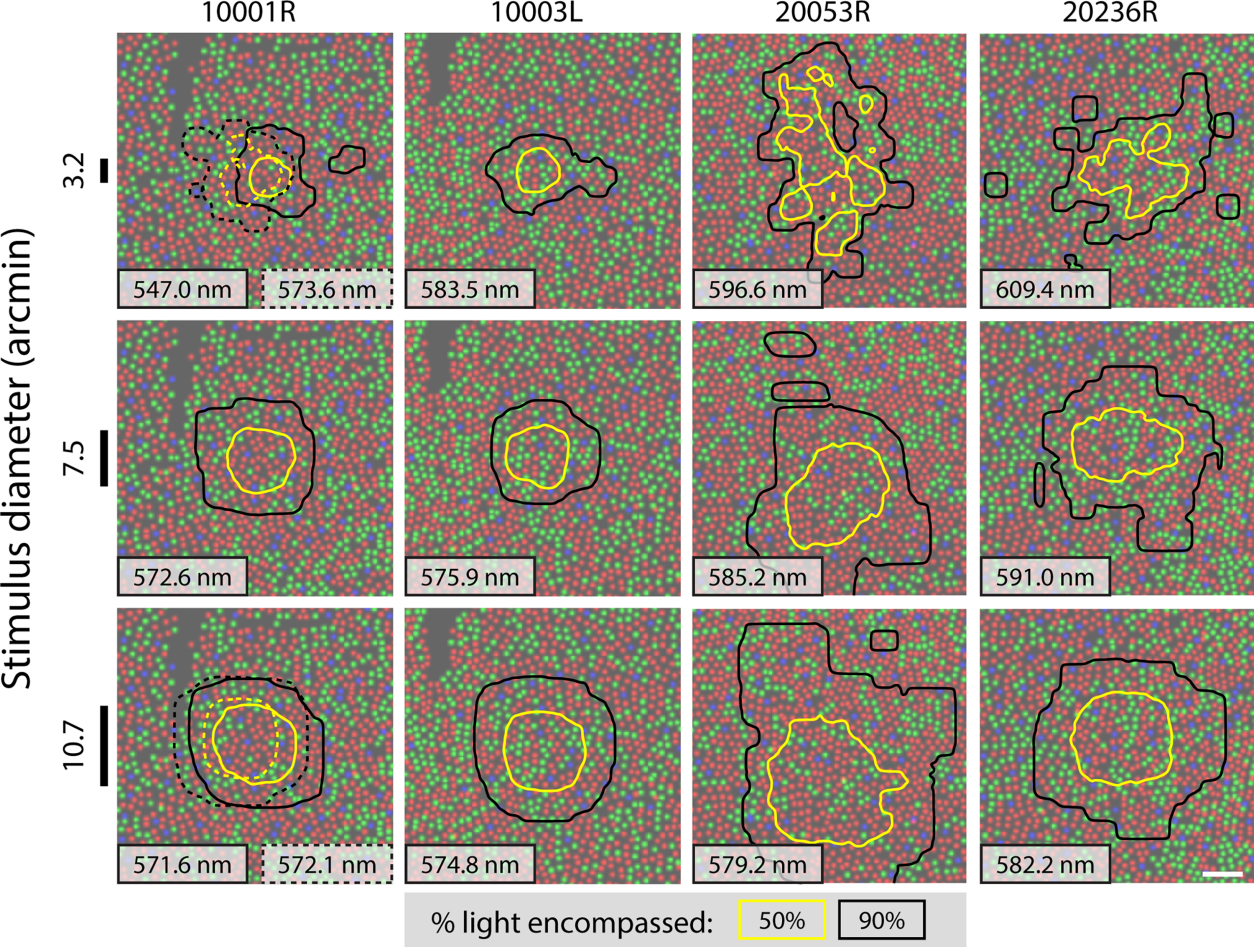
Light delivery contours for the four-frame stimuli. Yellow contours encircle 50% of the luminous power delivered across all trials; black contours encircle 90%. Columns correspond to different subjects, while rows correspond to different stimulus sizes. Scale bars indicating the stimulus diameter (black lines) are shown for each row. Unique yellow estimates corresponding to each condition are shown at the bottom of each retina map. Unique yellow measurements were made on two occasions for 10001R. The contours for the first measurement are solid and the ones for the second measurement are dashed. Scale bar in lower right panel indicates 5 arcmin of visual angle. Note: for subject 20236R, the middle-sized stimulus subtended 7.0 arcmin; these data are plotted with the 7.5 arcmin data obtained from the other participants.

In the parafoveal unique yellow measurements, stimuli were delivered to retinal locations where cone spectral types are known. Thus, for each trial, both the subject's response and the number of L-, M-, and S-cones hit by the stimulus were known. This allowed an analysis of the relationship between red/green color perception and exact cone topography that has historically not been possible in color psychophysics. We begin this analysis by computing the spatial distribution of light delivered to the retina across trials of a particular condition, for each subject. Figure 7 shows contours encompassing the central 50% and 90% of the retinal light distribution accumulated across all trials. These contours provide a visualization of how many L- and M-cones were stimulated for each condition and subject. From top to bottom, the rows correspond to stimuli with sizes of 3.2, 7.5, and 10.7 arcmin, all presented for four frames.

Due to fixational eye movements and diffraction blur, light is distributed over an area larger than the size of the stimulus across trials. Still, the contours make evident that fixation alone greatly aids in ensuring that stimuli are delivered to a confined, contiguous patch of retina. For the same subject and across conditions, contours are centered around similar points in the retina, which confirms that stimuli of different sizes and durations tended to be delivered to the same retinal loci. Unsurprisingly, contours increased in area with increasing stimulus size. Variability in contour shapes can be taken to represent the fixation stability of different subjects, which is known to vary considerably between individuals (Cherici, Kuang, Poletti, & Rucci, 2012).

The light delivery contours reveal that for one subject (10001R), a particularly L-cone-rich region of the retina was targeted. The 50% contour for the 3.2 arcmin condition (Figure 7, solid yellow line) encompasses almost entirely L-cones. Notably, this subject's unique yellow shifted to shorter wavelengths with decreased size for all durations except 15 frames. This is the opposite of the trend observed in our foveal data and in other participants’ parafoveal measurements.

#### Unique yellow versus proportion L

**Figure 8.**
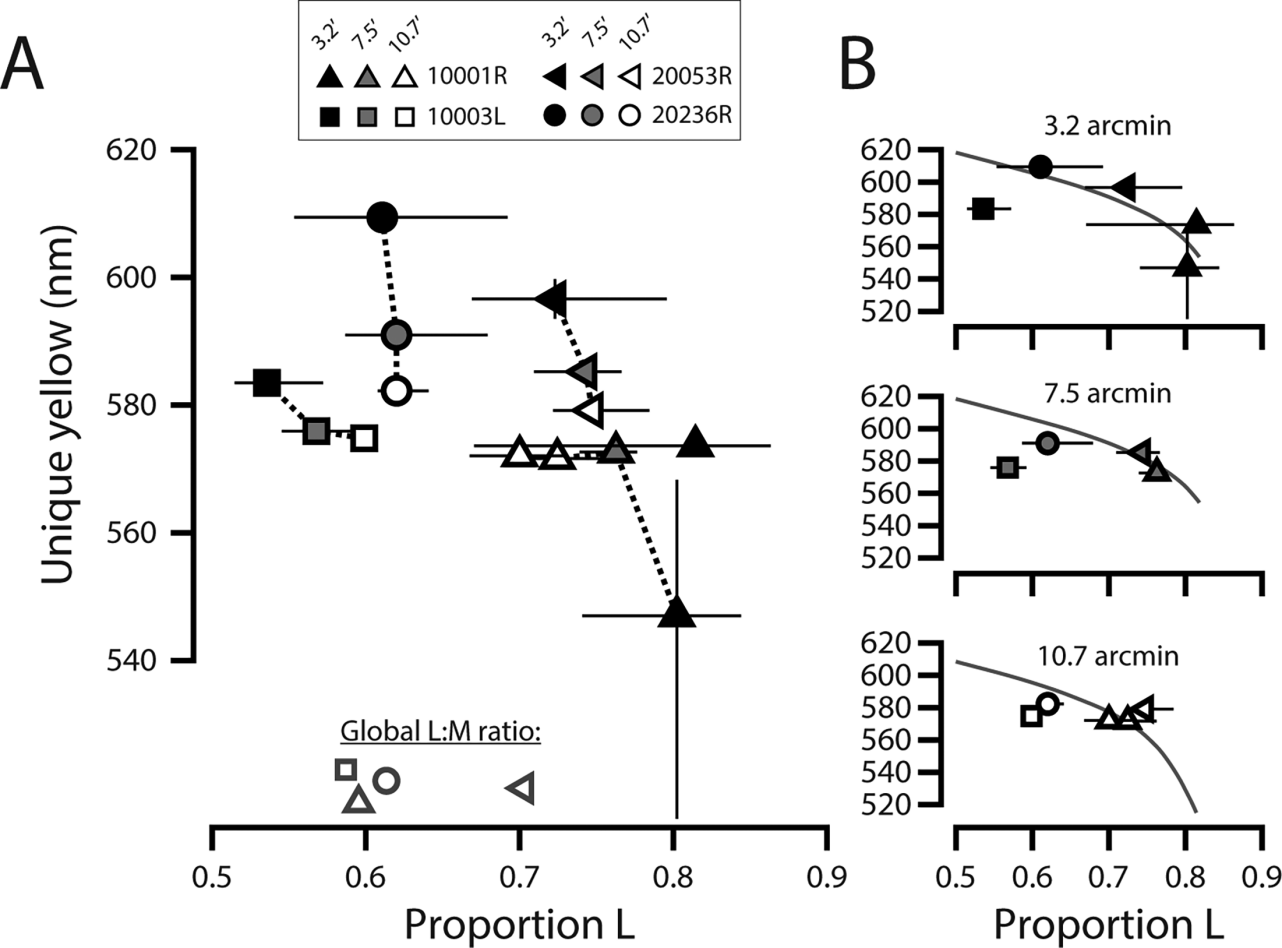
Dependence of unique yellow on L/M ratio. (**A**) Unique yellow estimates plotted against the median L-cone proportion of retinal locations stimulated across all trials. Black, gray, and white markers with black edges correspond to stimulus sizes of 3.2′, 7.5′, and 10.7′, respectively. Marker shapes correspond to different subjects, as indicated in the legend. Gray-edged markers near the abscissa indicate the global L/M ratios of each subject. Vertical errors bars are the 95% bootstrapped confidence intervals on each unique yellow estimate, while horizontal error bars give the interquartile range of local L-cone proportions encountered across trials. (**B**) The same data as in (**A**), separated by stimulus size and plotted in individual panels. In each panel, the solid curve gives the unique yellow values predicted by a simple model of L–M cone opponency described in the text. Note: for subject 20236R, the middle-sized stimulus subtended 7.0 arcmin; these data are plotted with the 7.5 arcmin data obtained from the other participants.

Measurements of unique yellow as a function of the proportion of L-cones (relative to the number of L- and M-cones) in the stimulated neighborhood are plotted in Figure 8. Figure 8A shows data from all subjects for the four-frame condition, and in Figure 8B, these values are replotted in separate panels according to stimulus size.

As suggested by Figure 8A, changes in proportion L did not predict changes in unique yellow uniformly across subjects. For subjects 20053R and 20236R, large changes in unique yellow accompanied miniscule changes in median local L/M ratio encountered by the stimulus as size is varied. In contrast, for 10003L and 10001R unique yellow was relatively stable over large changes in L-cone proportion engaged by the stimulus. This indicates that factors aside from cone topography must contribute to size-dependent changes in unique yellow. Still, for every subject, an increase in L/M ratio tended to pull unique yellow down to shorter wavelengths, although this effect was highly variable. Hence, it cannot be ruled out that within-subject variability in unique yellow depends at least in part on the relative numbers of L- and M-cones stimulated.

 In each panel of Figure 8B, the following model of cone-opponent ganglion cell activity (Otake & Cicerone 2000) was fit to the data:

$$\mathrm{Response}=\left(c-1\right)M\left(\lambda \right)-jL\left(\lambda \right).$$

Here, **M**(*λ*) and **L**(*λ*) are the M- and L-cone fundamentals, λ is the stimulus wavelength, *c* is the relative strength of the receptive field center, and *j* is the L/M ratio. The above expression represents the output of a foveal midget cell that receives excitatory input from a single M-cone and inhibitory input from both L- and M-cones. It is assumed that unique yellow is the wavelength of light, λ_UY_, that balances the relative L- and M-cone activities nulling such a cell as

$$\left(c-1\right)M\left(\lambda_{\mathrm{UY}}\right)-jL\left(\lambda_{\mathrm{UY}}\right)=0.$$

An optimal fit was found by computing the value of *c* that minimizes the mean squared error between the empirical data and the theoretical unique yellow versus proportion L function. The important feature of this model is that it predicts a shift in unique yellow toward longer wavelengths as L/M ratio decreases.

At larger stimulus sizes (7.5′ and 10.7′), between-subject variability in unique yellow seems lower than would be expected from the model, given the range of L/M ratios. At the smallest size (3.2′), unique yellow estimates are more variable between subjects, and this variability may bear some relation to the L-cone proportion encountered by the stimulus.

## Discussion

The central question of this work is how the wavelength of unique yellow changes as stimuli are restricted in space and time. Our results demonstrate that unique yellow varies with stimulus size and duration for small (<11 arcmin), briefly presented (<500 ms) flashes. At the fovea, unique yellow shifted to longer wavelengths with decreasing test size or duration, an effect that was observed to varying degrees in all our subjects (Figures 3 and 4). Our hue-scaling results suggest that small, brief flashes delivered to the fovea retained a yellowish appearance near the red–green equilibrium, although these stimuli were rated as less saturated than test spots metameric to longer or shorter wavelengths (Figure 5). In the parafovea, the wavelength of unique yellow became increasingly variable as stimulus size was reduced; these shifts did not exhibit a straightforward dependency on the underlying spectral topography of the cone mosaic (Figure 8). Below we discuss our findings in the context of previous work and consider the potential contributions of optical, receptoral, and post-receptoral factors to the trends we observed.

### Optical factors do not explain variance in small-spot unique yellow

Our foveal unique yellow measurements agreed closely with those of Otake and Cicerone (2000), although our methodologies differed (Figure 4). The earlier authors used monochromatic stimuli viewed through conventional optics, whereas our stimuli were mixtures of two narrowband primaries adjusted for chromatic aberration, corrected by adaptive optics and raster scanned onto the retina. The concordance between our data and those of Otake and Cicerone (2000) means that retinal light spread caused by high-order optical aberrations cannot explain the dependence of unique yellow on the nominal angular subtense of the stimulus.

It seems sensible that the mechanisms underlying color perception would not benefit from the correction of monochromatic, high-order aberrations. Color vision depends on a comparison of light absorbed by cones of different types arranged in an interleaved mosaic. Consequently, the circuits that implement those comparisons must operate on a coarser spatial scale than, for example, the visual pathways subserving high-resolution vision (Sekiguchi, Williams, & Brainard, 1993). While the correction of ocular aberrations can improve visual resolution up to the sampling limit of the cone mosaic (Rossi & Roorda, 2010; Yoon & Williams, 2002), it is likely that the information conveyed by more coarse-grained neural pathways is less affected by small optical imperfections (cf. Tuten et al., 2018).

If anything, previous work suggests circumventing retinal image degradation might be suboptimal for consistent color perception, particularly when stimulus size is small. Experiments using adaptive optics have shown that the color appearance of a monochromatic flash becomes more variable when the retinal profile of the stimulus is confined to the scale of individual photoreceptors (Hofer et al., 2005; Sabesan, Schmidt, Tuten, & Roorda, 2016; Schmidt, Boehm, Foote, & Roorda, 2018). In a similar vein, subtle chromatic aliasing can result when high spatial frequency achromatic gratings are viewed through well-focused optics, a perceptual artifact that may be attenuated under normal viewing conditions by the eye's imperfect optics (Williams, Sekiguchi, Haake, Brainard, & Packer, 1991; Zhang, Cottaris, & Brainard, 2022).

### Color appearance at red–green equilibrium

We used a hue-scaling procedure to quantify the appearance of a set of stimuli spanning the chromaticities utilized during the staircase procedure of our main experiment (Figure 2). Hue-scaling techniques have been applied to characterize the color appearance of visual stimuli, generating results from which inferences about the nature of postexcitation chromatic mechanisms have been drawn (Abramov, Gordon, & Chan, 1991; Bosten & Boehm 2014; De Valois, De Valois, Switkes, & Mahon, 1997; Emery, Volbrecht, Peterzell, & Webster, 2017). When stimulus diameter and duration were decreased, our hue-scaling results showed that perceived yellowness shifted to longer wavelengths (Figures 5A, 5B), a trend qualitatively similar to that observed in our staircase measurements of unique yellow (Figure 4) and those reported previously by Otake and Cicerone (2000).

These data also demonstrate that decreasing stimulus energy by reducing its duration or angular subtense led to an overall reduction in perceived saturation (Figure 5C). Color naming results from Weitzman and Kinney (1967) showed a similar trend, with the portion of the spectrum near unique yellow becoming increasingly desaturated as stimulus size was reduced from 54 to 21 to 11 arcmin. Under similar conditions to those reported here, Vanston, Boehm, Tuten, and Roorda (2023) found that the saturation of 543-nm AO-corrected flashes ranging in diameter from 0.5 to 4 arcmin depended on stimulus size in the parafoveal retina. Moreover, multiple studies have shown that reducing the stimulus duration of a small (∼10 arcmin) foveal field from ≥200 ms to ≤20 ms incurs a loss of saturation of middle-spectrum wavelengths, particularly those near 580 nm (Kaiser, 1968; Weitzman & Kinney, 1967). A similar trend is evident in the data obtained with our 1.1 arcmin stimulus delivered over increasingly smaller temporal windows (Figures 5C, 5D).

Despite the desaturation we observed with decreasing stimulus size and duration, the smallest, briefest stimulus judged by our subjects nonetheless retained perceived yellowness around the red–green equilibrium point (Figure 5). This result mirrors the findings of Ingling, Scneibner, and Boynton (1970), who used 3 arcmin, 75-ms flashes viewed foveally. Further, stimuli that appeared redder or greener tended to be rated as more saturated than lights metameric to intermediate (i.e., yellowish) wavelengths (Figure 5D). This is also consistent with previous color naming studies that employed small, brief flashes delivered to the fovea (Finkelstein & Hood, 1984; Ingling et al., 1970; Kaiser, 1968). It is tempting to attribute this dip in saturation around spectral yellow to the absence of S-cones in the central fovea (Curcio et al., 1991), as the tritanopic neutral point (i.e., the monochromatic wavelength tritanopic subjects confuse with white) typically falls between 570 and 580 nm; however, a similar notch in the saturation-versus-wavelength curve has been observed in measurements made in the parafoveal retina with spots large enough to ensure S-cones are involved in sampling the stimulus (Abramov et al., 1991; Abramov, Gordon, & Chan, 1992), suggesting post-receptoral factors may also contribute to this phenomenon.

### Spectral sampling by the trichromatic cone mosaic and unique yellow

We turn now to consider evidence that cone topography—specifically, the L/M-cone ratio encountered by the stimulus—may influence the locus of unique yellow under the conditions studied here. Although human color perception is fundamentally trichromatic, S-cones have negligible sensitivity to wavelengths above 550 nm (Stockman, Sharpe, & Fach, 1999), a portion of the visible spectrum that includes lights ordinarily perceived as green, yellow, orange, and red. This means that the red–green equilibrium wavelength will be determined by the relative contributions of the L- and M-cones to post-receptoral color channels. It has been suggested that if the L versus M signal that produces unique yellow corresponds to a fixed internal reference—either at the null point of a cone-opponent circuit or at some arbitrary reference level—then the wavelength that appears purely yellow would depend strongly on the relative number of L- and M-cones in the retina (Brainard et al., 2000; Otake & Cicerone, 2000). Alternatively, if color mechanisms instead self-calibrate to an external reference in order to compensate for variability introduced at the light encoding stage, then unique yellow may depend on the properties of the environment rather than L/M ratio (Brainard et al., 2000; Mollon, 2006; Mollon & Jordan, 1997; Neitz et al., 2002).

One of the advantages of the imaging and stimulation platform used in this study is that, when used in subjects whose cone spectral identities are known via optoretinography (Pandiyan et al., 2020), it enables hypotheses about the link between the spectral topography of the cone mosaic and visual perception to be interrogated with cellular-scale precision (Sabesan et al., 2016; Tuten, Harmening, Sabesan, Roorda, & Sincich, 2017). For the 10.7 arcmin stimulus, differences in unique yellow between subjects were small despite considerable differences in local L/M ratio stimulated (Figure 8B, bottom panel). This is consistent with previous work suggesting that unique yellow is independent of L/M ratio (Brainard et al., 2000; Mollon & Jordan, 1997; Neitz et al., 2002). An important methodological difference between these previous studies and the current one is the scale at which the link between cone demographics and color perception was assessed. For example, Neitz et al. (2002) compared global L/M ratio, estimated by full-field ERG flicker photometry, to large-stimulus (≥0.5°) unique yellow settings. Although the independence of unique yellow from L/M ratio is compatible with the idea that chromatic mechanisms calibrate themselves to compensate for intrinsic variability in spectral encoding, the relative coarseness of the measurements obscures whether this process is a global or local phenomenon.

A noteworthy feature of the trichromatic cone mosaic is that its constituents are arranged near-randomly (Hofer et al., 2005), resulting in local clusters of photoreceptors with L/M ratios that deviate from the global L/M ratio (Figure 1). If unique yellow is determined solely by the global L/M ratio, then the wavelength of unique yellow should vary most when stimuli are confined to pockets of the cone mosaic with skewed demographics. By contrast, if L/M ratio is compensated for locally, then unique yellow should exhibit little topographic variability, even when measured with small spots. In subjects 10001R and 20053R, the 10.7 arcmin stimulus was delivered to a retinal location with an L-cone proportion greater than their respective global averages (Figure 8A); however, both subjects converged on red–green equilibria that were very similar to those obtained from subjects 10003L and 20236R, where the stimulated cone neighborhoods were more in line with their respective population averages.

Compared with the 10.7 arcmin flash, the intersubject variation in unique yellow settings for the 3.2 arcmin flash was relatively large (Figure 8B, top panel). Data from this condition (in contrast with the other conditions) appear to fall closer to what would be predicted from a model in which L/M ratio determines unique yellow, where the red–green equilibrium shifts to longer wavelengths in retinal regions with comparatively few L-cones and vice versa (Brainard et al., 2000; Otake & Cicerone, 2000). In subject 10001R, an especially L-rich region of the parafoveal retina was tested on two separate occasions. The unique yellow measurements made with the 10.7 arcmin stimulus were consistent across sessions and, as mentioned above, were in close agreement with the settings made by the other participants (Figure 8B, up-facing triangles). However, due to a shift in fixation between the two visits on the order of a few cone diameters, the spectral neighborhoods engaged by the 3.2 arcmin stimulus varied substantially across sessions (Figure 7, top row, left column, yellow contours). Comparing the cone neighborhood interquartile ranges indicated by the horizontal lines in Figure 8B (upper panel, triangles), it is evident that the stimulus that more frequently encountered M-cones yielded a unique yellow estimate within the typical range recorded in the literature, whereas the session that engaged a more homogeneous patch of L-cones produced a unique yellow estimate that fell just a few nanometers above the green primary wavelength. In the latter case, the subject predominantly reported seeing red, even when the stimulus mixture contained primarily 543 nm light, underscoring the challenges the visual system faces when making inferences about color from a small number of univariant cone signals (Brainard, Williams, & Hofer, 2008; Schmidt, Boehm, Tuten, & Roorda, 2019).

Taken together, our results suggest the compensation for variations in L/M ratio across the parafovea may occur at a spatial scale bracketed by the smallest and largest stimuli used in this study. Neitz et al. (2002) measured changes in unique yellow after long-term exposure to altered spectral environments, an effect that exhibited interocular transfer. From these results, they concluded that the mechanisms involved in maintaining the stability of red–green equilibrium must reside in the cortex. Similarly, a recent neuroimaging study suggests that post-receptoral compensation for the diminished L versus M signal in anomalous trichromacy is evident in areas V2 and V3 but not area V1 (Tregillus et al., 2021). An advantage of a cortical locus for L/M ratio compensation is that the sites involved likely collect inputs from multiple cones, which may serve to enhance the reliability of the adaptation signal (Rieke & Rudd, 2009).

Finally, we note that the relative number of L-cones stimulated across trials cannot fully account for which wavelength was seen as purely yellow. First, although the data in Figure 8B are suggestive of a relationship between unique yellow and L/M ratio when spot size is small, more definitive conclusions could be drawn if the spectrum of local L/M ratios was sampled more evenly. This could be accomplished by using retinally stabilized stimuli to target multiple areas of interest in a single patch of classified retina; such measurements are a goal of future work. Moreover, in the two subjects who exhibited the largest unique yellow shifts with stimulus size (20236R and 20053R), the median relative number of L-cones illuminated (across trials) varied minimally between stimuli of different sizes (Figure 8A). Hence, a complete understanding of the processes underlying unique yellow requires both characterizing the effect of local spectral demographics on color perception and knowing the impact of manipulating the stimulus's spatiotemporal profile on the responses of post-receptoral mechanisms.

### Receptoral and post-receptoral factors

All in all, changes in local L/M ratio do not fully explain the variance of unique yellow with size and duration. Instead, an explanation must involve the intrinsic properties of cones and downstream circuitry. That smaller, briefer stimuli tend to look greener under the conditions used here, pushing unique yellow to longer wavelengths, may reflect spatiotemporal asymmetries in the neural pathways subserving hue perception. Incomplete knowledge about key aspects of color processing limits our ability to speculate on what these asymmetries are and how they might explain our data. Most importantly, the number and nature of chromatic channels responsible for color appearance are debated, and the neural substrates of such putative mechanisms have not been identified (Mollon, 2009). Additionally, the privileged status historically afforded to the unique hues has been called into question on the basis of behavioral and physiological evidence (Bosten & Boehm, 2014; Conway, Malik-Moraleda, & Gibson, 2023; Emery et al., 2017; Wool et al., 2015).

Despite the lack of consensus on how color information is organized in the brain, multistage color models typically assume that the subcortical cone-opponent pathways contribute to the appearance of unique hues (De Valois & De Valois, 1993; Stockman & Brainard, 2010; Wuerger, Atkinson, & Cropper, 2005). In a color space constructed from these cone-opponent dimensions, pure yellow plots at an oblique angle (∼310°) between the positive L–M and negative S – (L+M) axes (e.g., Webster et al., 2000). Thus, unique yellow corresponds to an increase in L-ON and S-OFF activity, or a decrease in M-OFF and S-ON activity, relative to an achromatic background. A shift in unique yellow toward longer wavelengths with reduced field size and exposure, as demonstrated in the present experiment, is equivalent to a rotation of unique yellow in cone-opponent space toward the positive L–M axis. This is consistent with a sensitivity loss in the L–M channel relative to the S – (L+M) channel. This could occur as early as the retina, or alternatively, a sensitivity loss in any higher-order mechanism tuned to a direction between our unique yellow estimates and the positive L–M axis could just as well explain a shift toward longer wavelengths (Webster & Mollon, 1994).

Asymmetries in spatiotemporal processing between the cone opponent channels could conceivably be the basis for unique yellow's size- and time-dependent shift. In the spatial domain, previous physiological and psychophysical work suggests that spatial convergence is generally greater in S-cone-driven pathways than in neurons tuned to the L–M axis (Field et al., 2010; Tailby, Solomon, & Lennie, 2008; Vassilev, Mihaylova, Racheva, Zlatkova, & Anderson, 2003; Volbrecht, Shrago, Schefrin, & Werner, 2000). Regarding the subcortical pathways that may influence the locus of unique yellow, if the S-OFF pathway sums input over a larger region than the L-ON pathway, then reductions in stimulus size should induce a larger reduction in the L-ON response relative to the S-OFF response. As noted above, this kind of sensitivity attenuation of the (L–M) channel, relative to the –S channel, would result in a shift of unique yellow to longer wavelengths. In terms of proposed higher-order mechanisms, the perceptive field for green (i.e., the stimulus size required for a certain percentage of greenness to be reported in hue-scaling studies) is larger than that for red (Abramov et al., 1991; Troup, Pitts, Volbrecht, & Nerger, 2005; Volbrecht, Clark, Nerger, & Randell, 2009). This suggests that the response of the neural process mediating the sensation of red falls faster than the response of the green process as stimulus size is reduced. Again, this type of asymmetric red/green spatial processing, which is also evident in older hue-scaling results (Weitzman & Kinney, 1969), predicts a red shift in unique yellow for increasingly small stimuli.

With regard to temporal processing, the impulse response of the S-cone pathway(s) has a higher latency and longer decay than that of the L–M pathway (Burr & Morrone, 1993; Shinomori & Werner, 2008; Smithson & Mollon, 2004). This may follow from the fact that S-cone photovoltages are more sluggish than those of L- and M-cones (Baudin, Angueyra, Sinha, & Rieke, 2019). Additionally, S-OFF signals are processed more slowly than S-ON signals (Shinomori & Werner, 2008). If, as stated earlier, the perception of unique yellow depends on joint –S and +(L–M) activity, then presumably there exists a downstream site at which signals from each of these chromatic channels interact. The output of this site depends on both the amplitudes of the inputs and their alignment in time (Cottaris & De Valois, 1998; Lee, Mollon, Zaidi, & Smithson, 2009). If very brief stimulus durations alter the relative phase or amplitude of the S-OFF and +(L–M) signals arriving at the combination site, this could, in principle, explain why unique yellow shifted to longer wavelengths when stimulus duration was reduced.

As summarized above, it is well established that the sensitivity of chromatic mechanisms depends strongly on stimulus spatiotemporal characteristics, with elevated thresholds generally observed with increasing spatial and/or temporal frequency (McKeefry, Murray, & Kulikowski, 2001; Mullen, 1985; Wuerger et al., 2020). Although all stimuli used in our study were presented at suprathreshold intensities, it is possible that a different pattern of results may have emerged if the test flashes were scaled to observers’ chromatic sensitivity at each combination of size and duration. In a conventional color display, the inputs to the chromatic mechanisms can, in principle, be manipulated without modulating activity in achromatic pathways. For the bichromatic increments used in this study, which contain both luminance and color information, equating detectability could only be achieved by adjusting stimulus luminance (i.e., the intensity of the red–green mixtures). Since the relative sensitivity of achromatic and chromatic pathways changes as stimulus size is reduced (Chaparro, Stromeyer, Kronauer, & Eskew, 1994; King-Smith & Carden, 1976), scaling our stimuli to detection threshold would not guarantee that the sensitivities of chromatic mechanisms were equated across conditions.

There are many other possible reasons for small, brief increments of yellowish light appearing different from larger, more sustained flashes, and to consider them all is beyond the scope of this article. Signals from rods, for example, can push unique yellow to longer wavelengths (Buck, Knight, & Bechtold, 2000; Buck, Thomas, Hillyer, & Samuelson, 2006). Ultimately, a full explanation of our data may involve complex aspects of neural color processing that are just beginning to be revealed by new techniques that enable circuit characterization at the scale of single cells in the retina (Kim et al., 2023; Wool, Packer, Zaidi, & Dacey, 2019) and at downstream sites (M. Li et al., 2022; P. Li, Garg, Zhang, Rashid, & Callaway, 2022; Liu et al., 2020; Nigam, Pojoga, & Dragoi, 2023). As our understanding of the machinery of color vision continues to evolve, the spatiotemporal dependencies of unique yellow observed in the present study may prove useful for evaluating neurobiologically inspired models aiming to link cone activations to color perception (cf. Rezeanu, Neitz, & Neitz, 2023; Schmidt, Touch, Neitz, & Neitz, 2016).

## Supplementary Material

Supplement 1

## Footnotes

1 Unlike many displays used in visual psychophysics, the AOSLO is a point-scanning device with complex temporal characteristics. Stimuli are rendered pixel by pixel on the retina via raster scanning, with each pixel illuminated for 50 ns. On a single display frame, the time it takes to render a stimulus therefore depends on its size. In the present study, most measurements were made using square stimuli with side lengths ranging from 1.1 to 10.7 arcmin; the single-frame rendering time for these small flashes ranges between ∼0.6 and ∼6.5 ms, respectively. Multiframe stimulation involves presenting the retina with a train of these brief flashes separated by the interframe interval of the AOSLO system (∼33.3 ms, in the present study). Because N-frame stimuli are separated by N – 1 stimulus intervals, the temporal display windows for the 4- and 15-frame stimuli were approximately 100 ms and 467 ms, respectively. For individual cones, the light exposure time per AOSLO frame was between 0.25 and 0.50 ms, depending on photoreceptor diameter, which varies across the eccentricities studied here.
