# Supplementary material for: Unique yellow shifts for small and brief stimuli in the central retina
Source: J Vis. 2024 Jun 4;24(6):2. doi: 10.1167/jov.24.6.2 (PMC11156209; doi:10.1167/jov.24.6.2)
Supplement: Supplement 1 [file jovi-24-6-2_s001.pdf]

Supplementary Information

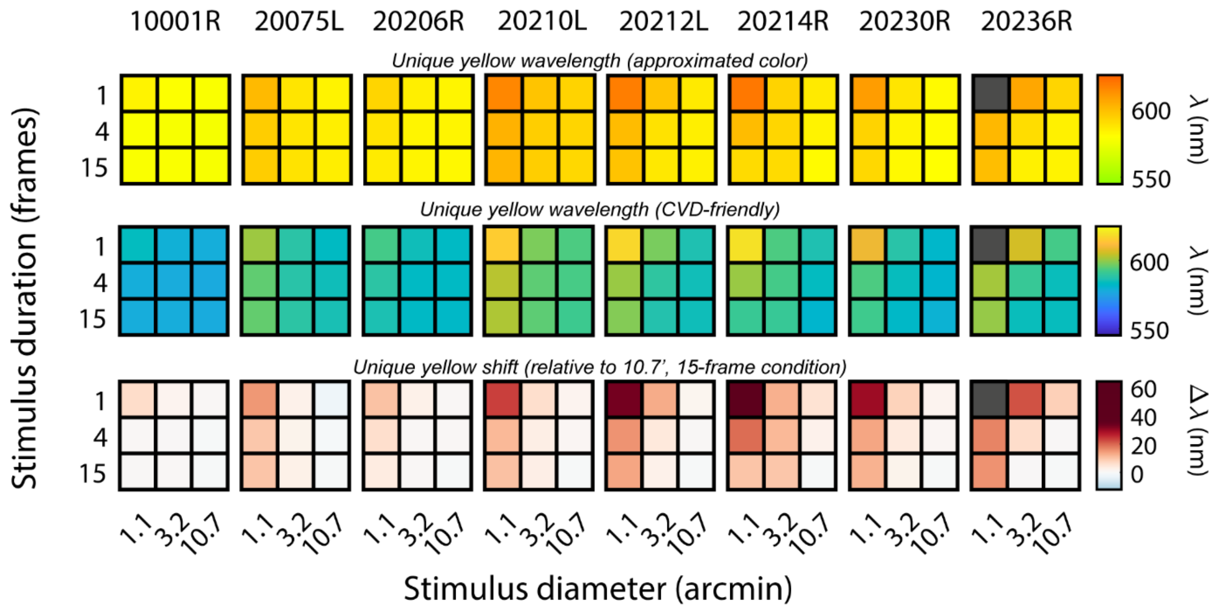

Figure S1 | Changes in foveal unique yellow measurements with changes in stimulus size in duration. (Top row) 3-by-3 matrices indicate the equivalent wavelength of unique yellow for each diameter-duration combination. Each square is color-coded to approximate the large-field appearance of the red-green equilibrium stimulus. Columns represent data from individual subjects. (Middle row) Unique yellow data are re-plotted using a perceptually uniform color map. (Bottom row) Changes in unique yellow with size and duration, plotted relative to the equilibrium wavelength estimated in each subject with the 10.7 arcmin, 15-frame stimulus (lower right box); positive values indicate a shift toward longer wavelengths. For all panels, black squares indicate stimulus-duration combinations for with the psychometric fitting did not yield a reliable unique yellow estimate.

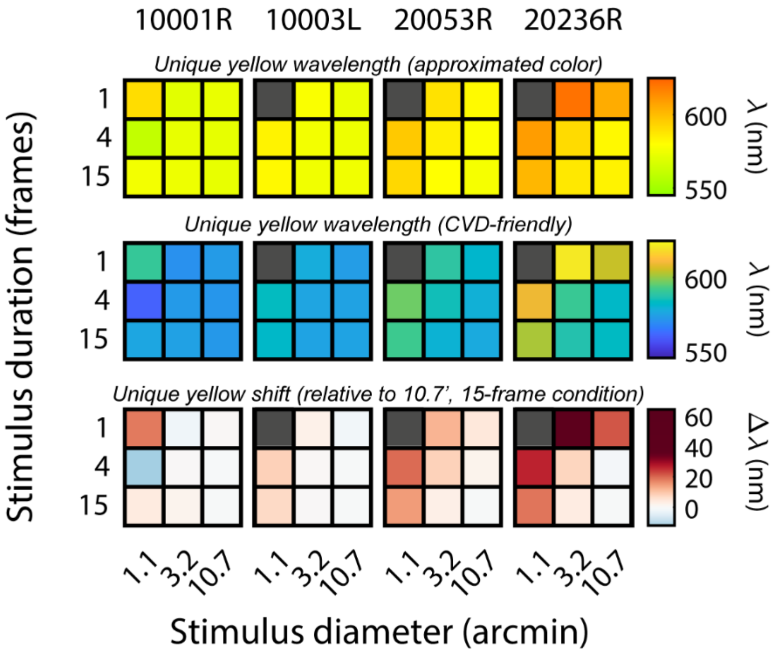

Figure S2 | Changes in parafoveal foveal unique yellow measurements with changes in stimulus size in duration. (Top row) 3-by-3 matrices indicate the equivalent wavelength of unique yellow for each diameter-duration combination. Each square is color-coded to approximate the large-field appearance of the red-green equilibrium stimulus. Columns represent data from individual subjects. (Middle row) Unique yellow data are re-plotted using a perceptually uniform color map. (Bottom row) Changes in unique yellow with size and duration, plotted relative to the equilibrium wavelength estimated in each subject with the 10.7 arcmin, 15-frame stimulus (lower right box) ; positive values indicate a shift toward longer wavelengths. For all panels, black squares indicate stimulus-duration combinations for with the psychometric fitting did not yield a reliable unique yellow estimate.
